# The Influence of Droplet Size and Emulsifiers on the In Vitro Digestive Properties of Bimodal Oil-in-Water Emulsions

**DOI:** 10.3390/foods14071239

**Published:** 2025-04-01

**Authors:** Takumi Umeda, Hiroyuki Kozu, Isao Kobayashi

**Affiliations:** Institute of Food Research, National Agriculture and Food Research Organization, 2-1-12 Kannondai, Tsukuba 305-8642, Ibaraki, Japan; umedat307@affrc.go.jp (T.U.); kozuh095@affrc.go.jp (H.K.)

**Keywords:** bimodal emulsion, monodisperse emulsion, nonionic emulsifier, citrus pectin, digestive properties, in vitro gastric digestion, in vitro intestinal digestion, digestion control

## Abstract

Lipids are often ingested via oil-in-water (O/W) emulsions, where interfacial properties and droplet size influence their digestibility. In this study, a bimodal O/W emulsion, termed Food Emulsion Blend (FEB), was prepared by mixing two monodisperse emulsions of different droplet sizes and compositions. The influence of droplet size and emulsifier type on in vitro digestion was evaluated. Soybean oil was used as the dispersed phase, and monodisperse emulsions were prepared via premix membrane emulsification using membranes with pore sizes of 1, 10, and 50 µm. Two selected emulsions were mixed in equal proportions to form FEB. The emulsifiers included 1.0% (*w*/*w*) Tween 20 (TW) or 0.5% (*w*/*w*) Tween 20 and 0.5% (*w*/*w*) citrus pectin (TWCP). The $d_{4,3}$ values of the emulsions stabilized by TW and TWCP ranged from 1.05 to 51.99 µm and from 1.19 to 46.94 µm, respectively. In vitro digestion revealed that all FEB samples retained bimodal size distributions post-gastric digestion. Free fatty acid release correlated strongly with the initial total droplet surface area for the TW- and TWCP-stabilized FEBs (R^2^ > 0.8). These results suggest that FEB allows for the precise control of lipid release, offering potential applications in food formulation.

## 1. Introduction

Lipids are one of the three major macronutrients (including carbohydrates and proteins) and have the highest energy density. Some bioactive compounds require an oil phase for encapsulation, suspension formulation, and absorption. Lipids are sources of essential fatty acid noncarriers of fat-soluble micronutrients, such as carotenoids [1]. In human diets, lipids are often consumed in oil-in-water (O/W) emulsions, such as soups and sauces, which also provide natural sources of fiber and micronutrients. Emulsions are colloidal systems in which the dispersed phase (oil) is finely distributed as droplets within a continuous aqueous phase [2]. When emulsified foods are ingested, they are rapidly swallowed, and the digestion process begins in the stomach and small intestine. In gastric and intestinal environments, oil droplets are partially digested by gastric fluids before entering the small intestine, where they undergo further hydrolysis by lipases and bile acids, leading to the release of free fatty acids (FFAs) and monoacylglycerol, which are subsequently absorbed by the body [3].

From the perspective of lipid digestion control in emulsions, interfacial properties are considered a critical factor [4]. Studies on controlling lipid digestibility through interfacial structures have demonstrated that the type and molecular weight of polymers adsorbed at the interface can regulate lipid bio accessibility [5,6]. Moreover, natural emulsifiers, such as pectins, proteins, and phospholipids, have attracted significant attention as the formation of protein-polysaccharide complex interfaces have been suggested to modulate lipid breakdown rates during digestion, offering potential applications in food matrix design [7]. Previous studies have primarily examined emulsions stabilized by single or combined emulsifiers. However, the extent to which a single emulsifier can control digestibility is often limited. In mixed-emulsifier systems, competitive adsorption and interactions during digestion introduce complexity, which challenges precise lipid digestion control.

Another key factor that influences lipid digestibility is particle size. When the droplet size of an emulsion decreases, the interfacial surface area available for lipase action increases, thereby accelerating lipid digestion [8]. Therefore, simultaneous control of both the ideal structure and droplet size distribution is expected to enable more precise regulation of lipid digestibility. However, several challenges remain in controlling droplet size distribution. Conventional emulsification devices, such as high-pressure and rotor-stator homogenizers and colloid mills, typically produce polydisperse emulsions, making precise digestion control based on droplet surface area difficult [9]. Recently, advances in emulsification technologies, such as membrane and microchannel emulsification, have enabled the production of monodisperse emulsions [10,11]. Nevertheless, achieving a specific droplet size requires the selection of appropriate membranes and the precise optimization of emulsification conditions, which pose challenges for widespread application in conventional food manufacturing processes.

A bimodal emulsion exhibits two distinct peaks in its droplet size distribution. Previous studies on bimodal emulsions have primarily focused on evaluating their rheological properties and storage stability [12,13,14,15]. Recently, a previous study reported the fabrication of bimodal emulsions with independent droplet size distributions by mixing two monodisperse emulsions of different sizes, followed by an evaluation of their flow properties [16]. We hypothesized that the use of bimodal emulsions with independent droplet size distributions could enable more precise control of lipid digestibility. Specifically, even at the same dispersed phase concentration, combining emulsions with different droplet sizes could allow for the regulation of the total interfacial area of oil droplets, thereby modulating the digestion rate. However, studies on the digestive characteristics of bimodal emulsions are scarce, and structural changes in droplets of different sizes and the mechanisms underlying lipid digestion in gastric and intestinal phases remain largely unexplored. Therefore, a systematic evaluation of the digestive properties of bimodal emulsions is necessary to advance our knowledge on precise lipid digestion control.

In this study, we propose a novel approach that integrates the control of both droplet size and interfacial structure, termed Food Emulsion Blend (FEB). FEB is a bimodal emulsion with an independent droplet size distribution obtained by blending two monodisperse emulsions with different droplet sizes and compositions. This blending process allows for precise control over droplet size distribution while maintaining the distinct properties of each monodisperse emulsion. Unlike conventional bimodal emulsions, FEB is designed to provide greater flexibility in selecting and combining emulsions to achieve specific functional properties. For example, by mixing emulsions with different droplet sizes, the total interfacial area of oil droplets can be adjusted at appropriate ratios while maintaining a constant oil-phase volume fraction in the system. The ability to customize both droplet size and composition suggests that FEB offers distinct advantages for food formulation and lipid digestion control. The proposed method allows for precise control of both the initial digestion rate and extent of lipid digestion. Moreover, the use of FEB may provide a simpler process for achieving stepwise digestion control, which is challenging with conventional monodisperse emulsions.

The objectives of this study were to fabricate FEBs with different droplet size combinations and emulsifier types and to elucidate their in vitro gastric and small intestinal digestive properties. In vitro digestion experiments were conducted to simulate gastric and intestinal digestion, and structural changes in the droplets at different digestion stages were evaluated.

## 2. Materials and Methods

### 2.1. Materials

In this study, polyoxyethylene (20) sorbitan monolaurate (Tween 20), a low-molecular-weight emulsifier, and citrus pectin (CP), a stabilizer, were used as model emulsifying and stabilizing agents, respectively. Refined soybean oil, a representative legume-derived lipid widely utilized as a food lipid source, was employed as the dispersed phase.

Tween 20, CP, refined soybean oil, Nile Red, sodium chloride (NaCl), 0.1 N sodium hydroxide solution (NaOH), 6 N hydrochloric acid (HCl), potassium dihydrogen phosphate (KH_2_PO_4_), potassium chloride (KCl), magnesium chloride hexahydrate (MgCl_2_(H_2_O)_6_), ammonium carbonate ((NH_4_)_2_CO_3_), sodium hydrogen carbonate (NaHCO_3_), and calcium chloride dihydrate (CaCl_2_·2H_2_O) were all purchased from FUJIFILM Wako Pure Chemical Co., Ltd. (Osaka, Japan). For digestive enzymes, pepsin from porcine gastric mucosa (P7000-1 Kg) and lipase from porcine pancreas were obtained from Sigma-Aldrich (St. Louis, MO, USA). Sodium taurodeoxycholate was purchased from Nacalai Tesque (Kyoto, Japan). Milli-Q water obtained from an ultrapure water purification system (Milli-Q IQ 7010; Merck, Darmstadt, Germany) was used for all experiments. Details of the composition of the emulsifiers and dispersed phase used in this study can be found in the Supplementary Materials (Table S1).

### 2.2. Preparation of Oil-in-Water Emulsions and Food Emulsion Blend

The continuous phase was prepared by dissolving a total of 1.0% (*w*/*w*) emulsifier in Milli-Q water. Tween 20 aqueous solution (TW) was prepared by dissolving 1.0% (*w*/*w*) Tween 20 in Milli-Q water under stirring at 25 °C for over 12 h. Additionally, a mixed solution of Tween 20 and CP (TWCP) was prepared by dissolving 0.5% (*w*/*w*) Tween 20 and 0.5% (*w*/*w*) CP in Milli-Q water under stirring at 80 °C for over 12 h. After stirring, all continuous phases were cooled to 25 °C, and the evaporated water was replenished with Milli-Q water to restore the exact weight, followed by additional stirring. For the dispersed phase, fluorescently labeled soybean oil was used. Nile Red was added to soybean oil at a concentration of 0.02% (*w*/*w*) and stirred under light-shielded conditions at 25 °C for over 12 h. Detailed physical properties of the prepared continuous and dispersed phases can be found in the Supplementary Materials (Table S2).

**Figure 1:**
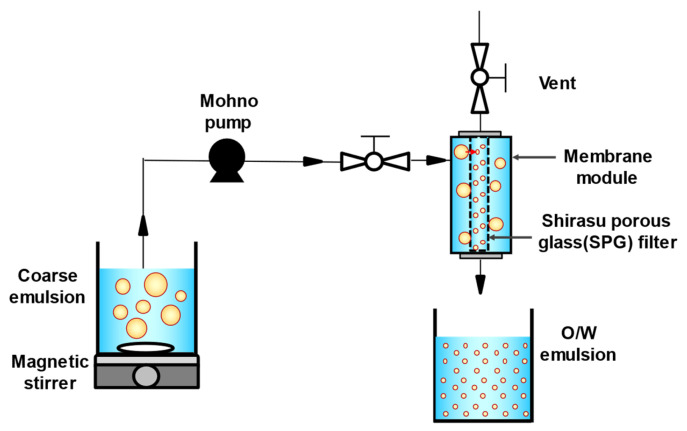
Schematic diagram of membrane emulsification apparatus and premix membrane emulsification. O/W, oil-in-water.

O/W emulsions with different droplet sizes (10% [*v*/*v*]) were prepared using a premix membrane emulsification method. Three types of tubular hydrophilic Shirasu porous glass (SPG) membranes (SPG Technology Co., Ltd., Miyazaki, Japan) with different pore sizes (1.1, 10.0, and 50.4 μm) were used. Prior to the experiment, membranes were immersed in the continuous phase to remove air and impurities and pre-cleaned using an ultrasonic cleaner (SND Co., Ltd., Nagano, Japan) at 38 kHz for 20 min. To prepare a coarse emulsion, continuous and dispersed phases were mixed using a magnetic stirrer (25 °C, 900 rpm, 1 min). The coarse emulsion was then passed through a membrane emulsification module equipped with an SPG membrane using a mohno pump (HEISHIN Ltd., Hyogo, Japan) to yield an O/W emulsion (Figure 1). The mean droplet sizes of all emulsions were comparable to the pore sizes of the SPG membranes used in the experiments. The emulsions were defined based on the combination of the continuous phase and SPG membrane pore size, as shown in Table 1. FEBs were prepared by mixing two emulsions of different droplet sizes in equal volumes. All droplets in the prepared emulsions and FEBs were fluorescently labeled.

### 2.3. In Vitro Gastric and Small Intenstinal Digestion Test

To evaluate the digestive properties of the prepared monodisperse emulsions and FEBs, in vitro gastric and intestinal digestion tests were conducted. Simulated gastric fluid (SGF) and simulated intestinal fluid (SIF) were prepared according to the compositions listed in Table 2 [17,18,19]. For the gastric digestion test, 15 mL of each incubated SGF (37 °C) and emulsion were mixed while stirring, and the pH was adjusted to 3.0. The mixture was then incubated in a shaking water bath (PERSONAL-11; TAITEC Co., Saitama, Japan) at 37 °C with 115 strokes/min for 2 h [20].

For the small intestinal digestion test, the gastric-digested sample was mixed with SIF at a 1:1 ratio and adjusted to pH 7.0. In vitro intestinal digestion was performed at 37 °C for 120 min using the pH-stat method. This method was used to monitor decreases in pH caused by the release of FFAs during lipid hydrolysis by lipases. The pH was maintained at 7.0 through titrating NaOH using a potentiometric automatic titrator (AT-710; Kyoto Electronics Manufacturing Co., Ltd., Kyoto, Japan), allowing for evaluations of the lipid digestion rate and extent [21]. The titrated volume of NaOH was recorded every 3 min throughout the intestinal digestion test.

### 2.4. Measurements and Analysis

#### 2.4.1. Droplet Size Distribution

The droplet and particle size distribution of the prepared O/W emulsions and FEBs were measured using a laser diffraction particle size analyzer (LS13 320; Beckman Coulter, Brea, CA, USA). Particle size distribution was measured using the intensity differential scattering (PIDS) method. The PIDS method enables the accurate measurement of submicron particles by analyzing the scattering intensity at multiple polarization angles. The PIDS technique enhances the size distribution analysis, particularly for emulsions containing droplets in the submicron range. This technique is also advantageous for measuring a wide range of particle sizes, particularly larger droplets, due to its ability to detect scattered light at multiple wavelengths. In contrast, dynamic light scattering provides a higher resolution for smaller nanoparticles but may be less accurate for polydisperse systems. Given the broad size distribution of FEBs, PIDS was selected as the optimal method for droplet size analysis.

Measurements were performed in triplicate at 25 °C, and the mean value was used for analysis. The mean droplet size was determined as the volume-weighted mean diameter ($d_{4,3}$) and calculated using the following equation:

(1) $$d_{4,3}=\sum n_{i}{d_{i}}^{4}/\sum n_{i}{d_{i}}^{3}$$

where $d_{i}$ represents the diameter of the i droplet and n represents the number of droplets measured (n = 100). Each measurement was performed three times, and the mean value was used for analysis. The relative span factor (RSF), which represents the uniformity of the droplet size distribution, was calculated using the following equation:

(2) $$RSF=\frac{d_{90}-d_{10}}{d_{50}}$$

where $d_{10}$, $d_{50}$, and $d_{90}$ correspond to the droplet sizes at which 10%, 50%, and 90% of the total volume of droplets are included in the cumulative size distribution graph, respectively.

#### 2.4.2. Microscopic Observation

Fluorescence microscopy (BZ-8100; Keyence, Osaka, Japan) was used to observe the emulsions immediately after preparation and at each digestion stage. All samples were examined using a 10× objective lens, and the stained droplets were visualized using a BZ filter TRITC (excitation wavelength: 540/525 nm; emission wavelength: 605/655 nm; dichroic mirror wavelength: 565 nm).

#### 2.4.3. Zeta Potential

The electrostatic charge of the dispersed droplets is a key parameter that influences emulsion stability and can provide insights into the interfacial state of emulsions during digestion. The zeta potential of the droplets was measured using a Zetasizer Nano ZS (Malvern Panalytical Ltd., Malvern, UK) for both freshly prepared emulsions and samples at different digestion stages. The samples were diluted by more than 50-fold with Milli-Q water before being injected into a capillary cell, and voltage was applied according to the measurement protocol. The zeta potential was determined based on the electrophoretic mobility calculated from the intensity of light scattered by particles due to Brownian motion. Measurements were performed in triplicate at 25 °C, and the mean value was used for analysis.

#### 2.4.4. Calculation of Free Fatty Acid Release

During digestion, lipases in the SIF hydrolyzes triacylglycerols, releasing FFAs. Each triacylglycerol molecule produces two FFA molecules upon complete digestion. Therefore, the percentage of FFAs released can be calculated based on the total amount of triacylglycerols present in the digestion cell and the amount of FFAs released at a specific digestion time [22]. The final FFA release rate after 120 min of digestion was determined using the following equation:

(3) $$FFA\left(\%\right)=\frac{V_{NaOH}\times {\;M}_{NaOH}\times M_{Lipid}}{2W_{Lipid}}\times 100$$

where $V_{NaOH}$ is the volume of NaOH titrated (L) at each time point, $M_{NaOH}$ is the molar concentration of the NaOH solution (M), $W_{Lipid}$ is the total mass of oil present before digestion (g), and $M_{Lipid}$ is the molecular weight of the lipid (g mol^−1^).

### 2.5. Statistical Analysis

All experiments were conducted independently and measured in triplicate. Statistical analysis of experimental data was performed using R software v4.2.3 (https://www.r-project.org/, Access date: 27 December 2024) with the EZR v1.61 plugin (Saitama Medical Center, Jichi Medical University, Shimotsuke, Japan) applied [23]. Statistical significance was determined using Tukey’s test and Student’s *t*-test, with significance set at *p* < 0.05.

## 3. Results and Discussion

### 3.1. Initial Phase

#### 3.1.1. The Characteristics of the Freshly Prepared Emulsions and Food Emulsion Blends

**Figure 2:**
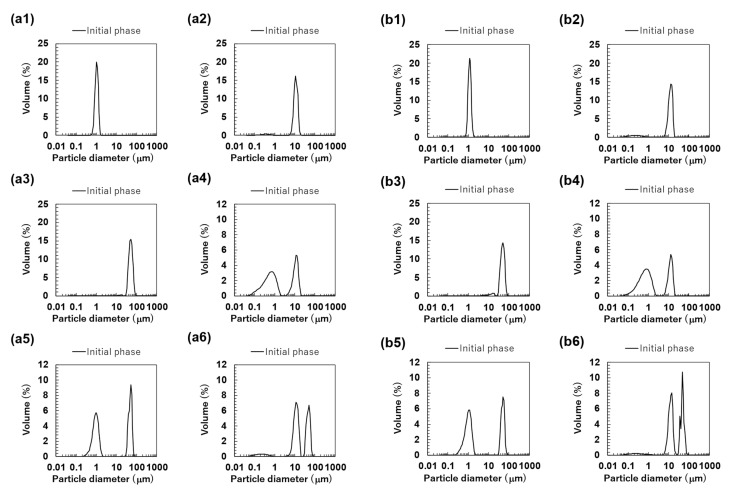
The particle size distributions of the emulsions and Food Emulsion Blends (FEBs) at the initial phase. All results were obtained using a particle size analyzer. Monodisperse emulsions stabilized by Tween 20 (TW): (**a1**) TW_1_, (**a2**) TW_10_, and (**a3**) TW_50_; FEBs stabilized by TW: (**a4**) TW_1,10_, (**a5**) TW_1,50_, and (**a6**) TW_10,50_; monodisperse emulsions stabilized by TW and citrus pectin (TWCP): (**b1**) TWCP_1_, (**b2**) TWCP_10_, and (**b3**) TWCP_50_; FEBs stabilized by TWCP: (**b4**) TWCP_1,10_, (**b5**) TWCP_1,50_, and (**b6**) TWCP_10,50_.

**Figure 3:**
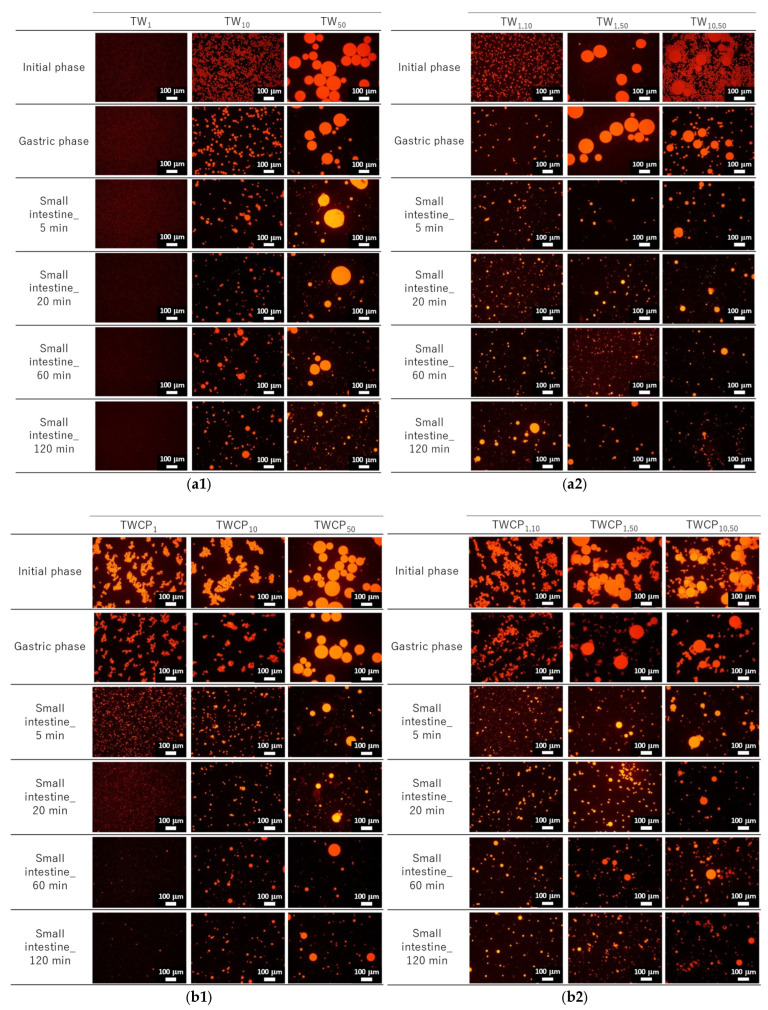
Fluorescence micrographs at the initial, gastric digestion, and intestinal digestion stages (5, 20, 60, and 120 min). Oil droplets were stained with Nile Red. Emulsions (TW_1_, TW_10_, and TW_50_) (**a1**) and Food Emulsion Blends (FEBs) (TW_1,10_, TW_1,50_, and TW_10,50_) (**a2**) stabilized by Tween 20 (TW). Emulsions (TWCP_1_, TWCP_10_, and TWCP_50_) (**b1**) and FEBs (TWCP_1,10_, TWCP_1,50_, and TWCP_10,50_) (**b2**) stabilized by TW and citrus pectin (TWCP).

**Figure 4:**
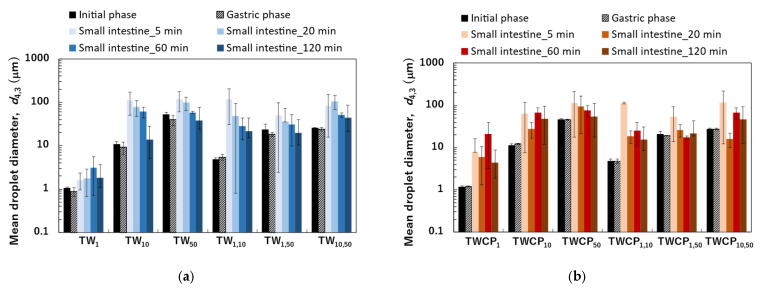
The mean droplet diameter measured at the initial, gastric, and intestinal digestion stages (5, 20, 60, and 120 min). The mean diameter was expressed as the volume-weighted mean diameter ($d_{4,3}$). (**a**) Emulsions and Food Emulsion Blends (FEBs) stabilized by Tween 20 (TW); (**b**) emulsions and FEBs stabilized by TW and citrus pectin (TWCP).

The particle size distributions of the O/W emulsions and FEBs prepared using TW as the continuous phase, as measured via a particle size analyzer, are shown in Figure 2a. The particle size distributions of TW_1_, TW_10_, and TW_50_ were confirmed to be monodisperse (Figure 2(a1–a3)). Fluorescence microscopy confirmed that all droplets remained stable without aggregation (Figure 3(a1)). The $d_{4,3}$ values of TW_1_, TW_10_, and TW_50_ were 1.05, 10.98, and 51.99 μm, respectively, with RSF values of 0.09, 0.47, and 0.17, respectively (Figure 4a). The mean droplet size of all monodisperse emulsions was within the range of 1.0–1.1 times the pore size of the SPG membranes used, indicating that the premix membrane emulsification method effectively controlled the droplet size. The mean droplet sizes of the emulsions and FEBs immediately after preparation can be found in the Supplementary Materials (Table S3).

The particle size distribution of freshly prepared FEBs (TW_1,10_, TW_1,50_, and TW_10,50_) exhibited a distinct bimodal distribution regardless of the droplet size combination (Figure 2(a4–a6)). The $d_{4,3}$ values of TW_1,10_, TW_1,50_, and TW_10,50_ were 4.86, 23.63, and 25.64 μm, respectively (Figure 4a). The mode diameters of the two peaks in each FEB were analyzed, revealing values of 0.72 and 11.83 μm for TW_1,10_; 0.95 and 52.62 μm for TW_1,50_; and 11.83 and 52.62 μm for TW_10,50_. As the mode diameters of TW_1_, TW_10_, and TW_50_ before mixing were 1.05, 10.78, and 52.62 μm, respectively, those of the two peaks in the FEBs were consistent with those of the original monodisperse emulsions.

**Figure 5:**
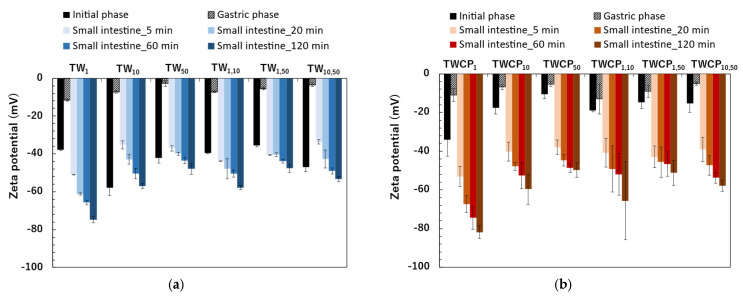
The zeta potential of the emulsions and Food Emulsion Blends (FEBs) at the initial and digestion stages. (**a**) The emulsions and FEBs stabilized by Tween 20 (TW); (**b**) the emulsions and FEBs stabilized by TW and citrus pectin (TWCP).

The zeta potentials of the TW emulsions and FEBs were negative immediately after preparation (Figure 5a). The zeta potentials ranged from −35.5 to −58.0 mV. The zeta potentials of the FEBs were intermediate between those of the two original emulsions before mixing. The zeta potentials of the emulsions and FEBs immediately after preparation can be found in the Supplementary Materials (Table S4). Tween 20 is known as a nonionic emulsifier and is generally expected to be uncharged. However, in this study, the emulsions stabilized by Tween 20 exhibited a negative charge. This phenomenon has also been widely reported in previous studies [24,25,26]. Additionally, research investigating this phenomenon suggests that the negative charge observed in emulsions stabilized by Tween 20 may be attributed to the preferential adsorption of hydroxide ions (OH^−^) from water onto the oil droplet surface or the presence of FFA impurities in the surfactant [2,27].

The particle size distribution of the O/W emulsions and FEBs prepared using TWCP as the continuous phase is shown in Figure 2b. TWCP_1_ exhibited a single peak, confirming that it is a monodisperse emulsion (Figure 2(b1)). TWCP_10_ and TWCP_50_ showed slight additional peaks at approximately 0.2 and 10 μm, respectively; however, both accounted for less than 1% of the total volume and were thus negligible (Figure 2(b2,b3)). Fluorescence microscopy revealed droplet aggregation in the TWCP emulsions, which was not observed in the particle size distribution measurements (Figure 3(b1)). The $d_{4,3}$ values of TWCP_1_, TWCP_10_, and TWCP_50_ were 1.19, 11.37, and 46.94 μm, respectively, with RSF values of 0.09, 0.50, and 1.01, respectively (Figure 4b).

For TWCP_1,10_, TWCP_1,50_, and TWCP_10,50_, two droplet populations of different sizes aggregated irregularly, similar to what was observed for the emulsions before mixing (Figure 3(b2)). However, their particle size distributions exhibited distinct bimodal peaks (Figure 2(b4–b6)). The $d_{4,3}$ values of TWCP_1,10_, TWCP_1,50_, and TWCP_10,50_ were 4.88, 21.32, and 27.50 μm, respectively (Figure 4b). The mode diameters of the two peaks in each FEB were 0.87 and 12.99 μm for TWCP_1,10_; 1.15 and 52.62 μm for TWCP_1,50_; and 14.26 and 47.94 μm for TWCP_10,50_. As the mode diameters of TWCP_1_, TWCP_10_, and TWCP_50_ before mixing were 1.15, 12.99, and 52.62 μm, respectively, the two peaks observed for the TWCP-based FEBs were consistent with those of the original monodisperse emulsions.

The zeta potentials of the freshly prepared emulsions and FEBs stabilized by TWCP were also negative, similar to that of the TW emulsions (Figure 5b). The zeta potentials ranged from −10.6 to −34.0 mV. In the TWCP-stabilized emulsions, smaller droplet sizes exhibited higher absolute zeta potential values.

#### 3.1.2. Microstructure of Emulsions and Food Emulsion Blends at Initial Phase

The emulsions prepared using TW and TWCP as the continuous phase exhibited monodisperse distribution in all cases. Furthermore, the FEBs produced by mixing these monodisperse emulsions exhibited two distinct peaks in their droplet size distribution regardless of the droplet size combination used. Previous studies on bimodal emulsions with independent droplet size distributions have primarily focused on analyzing the rheological properties of such emulsions with high dispersed-phase volume fractions [16]. However, the results of this study demonstrate the ability to produce bimodal emulsions with a lower dispersed-phase concentration.

The particle size distributions of the FEBs containing 1 μm droplets (TW_1,10_, TW_1,50_, TWCP_1,10_, and TWCP_1,50_) were broader than those of TW_1_ at the initial stage (Figure 2(a4,a5,b4,b5)). However, the microscopic observations revealed that submicron-sized microdroplets were not present in any of the samples (Figure 3(a2,b2)). The reason for the broader droplet size distribution observed in the FEBs mixed with 1 μm emulsions compared with that of their original monodisperse emulsions remains unclear; however, this is presumed to be due to specifications of the particle size analyzer.

In the emulsions and FEBs stabilized by TWCP, droplet aggregation was observed at the initial stage; however, this did not affect the particle size distribution measurements. This suggests that the aggregates formed by CP were likely dispersed by the convective flow within the particle size analyzer. Understanding the interfacial state of droplets is critical for emulsions stabilized by multiple emulsifiers. Previous studies have suggested that nonionic emulsifiers, such as Tween 80, strongly adsorb to the oil droplet interface, whereas CP primarily disperses on the droplet surface or within the continuous phase [28,29]. Owing to its high molecular weight and numerous carboxyl groups, CP readily forms crosslinked structures on the droplet surface [30]. In the emulsions prepared in the present study with both CP and Tween 20, a majority of the pectin was likely dispersed in the continuous phase or on the droplet surface, whereas a portion was likely present at the oil droplet interface along with Tween 20. This weak crosslinked structure may have contributed to preventing droplet collisions while also promoting aggregate formation.

The absolute zeta potential was lower in the TWCP-stabilized samples than in the TW-stabilized ones. This result suggests that competitive adsorption between Tween 20 and CP at the interface reduced electrostatic repulsion compared with that in emulsions stabilized with Tween 20 alone. In addition, the formation of crosslinked structures involving carboxyl groups in CP may have contributed to this effect.

### 3.2. Gastric Phase

#### 3.2.1. Emulsions and Food Emulsion Blends After Gastric Digestion

**Figure 6:**
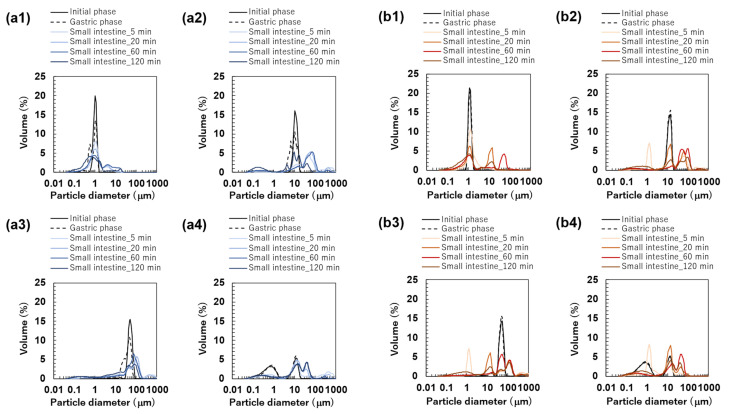

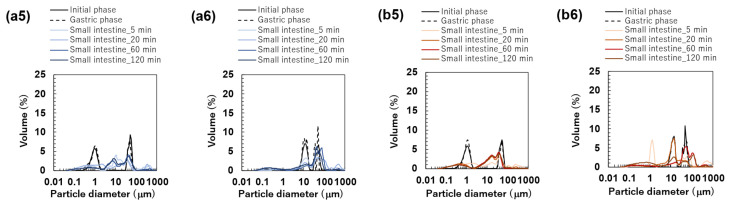
Particle size distribution of emulsions at the initial phase and each digestion phase. All results were obtained using particle size analyzer. Emulsions stabilized by Tween 20 (TW): (**a1**) TW_1_, (**a2**) TW_10_, and (**a3**) TW_50_; Food Emulsion Blends (FEBs) stabilized by TW: (**a4**) TW_1,10_, (**a5**) TW_1,50_, and (**a6**) TW_10,50_; emulsions stabilized by TW and citrus pectin (TWCP): (**b1**) TWCP_1_, (**b2**) TWCP_10_, and (**b3**) TWCP_50_; FEBs stabilized by TWCP: (**b4**) TWCP_1,10_, (**b5**) TWCP_1,50_, and (**b6**) TWCP_10,50_.

The particle size distributions of the emulsions and FEBs stabilized by TW during the gastric and intestinal digestion phases are shown in Figure 6a. The particle size distribution of monodisperse emulsions stabilized by TW during the gastric digestion phase largely overlapped with that observed in the initial phase regardless of droplet size (Figure 6(a1–a3)). Fluorescence microscopy revealed that TW_1_ and TW_10_ did not exhibit coalescence or droplet fragmentation, indicating their stability even under low-pH conditions in the stomach (Figure 3(a1)). In contrast, TW_50_ exhibited an increase in the proportion of 20 μm droplets, leading to a broader peak in size distribution. The $d_{4,3}$ values of TW_1_, TW_10_, and TW_50_ after gastric digestion were 0.88, 9.23, and 39.14 μm, respectively, with RSF values of 0.15, 0.47, and 1.24, respectively (Figure 4a).

All FEBs stabilized by TW maintained their independent biphasic droplet size distributions even after gastric digestion (Figure 6(a4–a6)), consistent with the fluorescence microscopy observations (Figure 3(a1)). The mode diameters of the two peaks observed in TW_10,50_ after gastric digestion were 14.26 and 47.94 μm, indicating their slight convergence compared with that during the initial phase. However, the mode diameters in TW_1,10_ (0.79 and 11.83 μm) and TW_1,50_ (1.05 and 47.94 μm) remained almost unchanged. The mean droplet size of the emulsions and FEBs after gastric digestion can be found in the Supplementary Materials (Table S3).

After gastric digestion, the absolute values of the zeta potentials of the emulsions and FEBs stabilized by TW significantly decreased to near the isoelectric point, ranging from −2.8 to −11.5 mV (Figure 5a). Among the samples, TW_1_ exhibited the highest absolute zeta potential, whereas TW_50_ had the lowest. The zeta potentials of the FEBs after gastric digestion were intermediate compared to those of the two monodisperse emulsions. The zeta potentials of the emulsions and FEBs after gastric digestion can be found in the Supplementary Materials (Table S4).

The particle size distributions of the emulsions and FEBs stabilized by TWCP during gastric and intestinal digestion are shown in Figure 6b. Similarly to the initial phase, the monodisperse emulsions stabilized by TWCP formed aggregates during the gastric digestion phase; however, the aggregate size decreased (Figure 3(b1)). This result suggests that pH conditions and shaking during in vitro gastric digestion partially disrupted the crosslinked CP. However, droplet coalescence and fragmentation were not observed. The particle size distributions after gastric digestion remained nearly identical to those observed in the initial phase regardless of droplet size (Figure 6(b1–b3)). The $d_{4,3}$ values of TWCP_1_, TWCP_10_, and TWCP_50_ were 1.22, 12.31, and 45.59 μm, respectively, with RSF values of 0.06, 0.57, and 0.74, respectively (Figure 4b).

Similarly, to monodisperse emulsions, the aggregates in FEBs stabilized by TWCP decreased in size after gastric digestion (Figure 3(b2)). In TWCP_1,50_ and TWCP_10,50_, aggregates were formed where smaller droplets adsorbed onto the interface of larger droplets. Regardless of the droplet size combination, all FEBs retained their biphasic size distributions after gastric digestion, with no peak shifts or emergence of new droplet size peaks observed. The $d_{4,3}$ values of TWCP_1,10_, TWCP_1,50_, and TWCP_10,50_ were 4.77, 19.23, and 27.49 μm, respectively (Figure 5b). The mode diameters of the two peaks in the FEBs stabilized by TWCP were nearly the same as those observed during the initial phase. After gastric digestion, the mode diameters of TWCP_1,10_, TWCP_1,50_, and TWCP_10,50_ were 0.87 and 12.99 μm; 1.15 and 52.62 μm; and 12.99 and 52.62 μm, respectively.

The absolute values of the zeta potential in the TWCP-stabilized samples also decreased to near the isoelectric point after gastric digestion, similar to those observed for the TW-stabilized samples (Figure 5b). The zeta potentials of the emulsions and FEBs stabilized by TWCP after gastric digestion ranged from −5.3 to −13.0 mV.

#### 3.2.2. Analysis of Droplet Stability During Gastric Digestion

The emulsions stabilized by TW and TWCP exhibited high stability during gastric digestion. Tween 20, a nonionic emulsifier, is known for its strong resistance to acidic conditions [28]. In our study, Tween 20, which has a high emulsifying capacity and acid stability, was adsorbed onto the oil droplet interface, preventing coalescence in monodisperse emulsions stabilized by TW or TWCP, even under low pH conditions in the stomach. The $d_{4,3}$ values of the emulsions stabilized by TW after gastric digestion decreased compared with those measured during the initial phase (Figure 4a). The droplet sizes of TW_10_ and TW_50_ used in this study were larger than those of the emulsions used in previous studies [31,32]. Therefore, even with acid-stable TW, some droplet refinement occurring due to shear forces can be inferred.

In contrast, the $d_{4,3}$ values of the emulsions stabilized by TWCP after gastric digestion remained unchanged compared with those measured during the initial phase. The enhanced stability of TWCP-stabilized emulsions compared with that of TW-stabilized emulsions can be attributed to the increase in viscosity of both the continuous phase and emulsion due to CP addition (Figures S1 and S2).

The FEBs stabilized by TW and TWCP retained independent biphasic size distributions until the gastric digestion phase regardless of the emulsifier type or droplet size combination used. Generally, polydisperse emulsions such as bimodal ones are more susceptible to droplet destabilization under physical stress [33]. Although it was anticipated that the FEBs might undergo coalescence due to physical and chemical stresses, such as shaking and low pH conditions, the results of this study demonstrated that using Tween 20 allowed the FEBs to maintain their independent biphasic size distributions even after gastric digestion.

After gastric digestion, the two peaks in the size distribution of FEBs stabilized by TW exhibited a slight tendency to converge compared with those observed during the initial phase. In contrast, the FEBs stabilized by TWCP did not exhibit this tendency. These results suggest that the presence of low concentrations of CP near the oil droplet interface suppressed droplet contact caused by shear forces in the stomach, further enhancing stability during the gastric digestion phase.

### 3.3. Small Intenstinal Digestion Phase

#### 3.3.1. Emulsions and Food Emulsion Blends During Small Intestinal Digestion Phase

Particle size distribution measurements and microscopic observations revealed that all monodisperse emulsions lost their monodispersity within 5 min after the start of the intestinal digestion phase (Figure 3(a1) and Figure 6(a1–a3)). The main peak of TW_1_ remained at approximately 1 μm even after intestinal digestion. In contrast, the main peaks of TW_10_ and TW_50_ shifted to 50–100 μm within 5 min after digestion. Throughout the entire digestion period, the $d_{4,3}$ values of TW_1_ showed minimal variation, ranging from 1.64 to 3.11 μm (Figure 4a). Conversely, the $d_{4,3}$ values of TW_10_ and TW_50_ rapidly increased within 5 min of digestion and subsequently decreased over time; the $d_{4,3}$ values at 5 min for TW_10_ and TW_50_ were 110.32 and 118.36 μm, respectively, whereas at 120 min, they decreased to 13.90 and 38.44 μm, respectively. The mean droplet size of the emulsions and FEBs during intestinal digestion can be found in the Supplementary Materials (Table S3).

All FEBs stabilized by TW lost their independent biphasic size distributions during the intestinal digestion phase (Figure 3(a2) and Figure 6(a4–a6)). After 120 min of intestinal digestion, TW_1,10_ and TW_1,50_ exhibited biphasic distributions with peaks at approximately 10 and 40 μm, respectively, showing similar waveforms. In contrast, the size distribution of TW_10,50_ after 120 min of digestion differed from that of TW_1,10_ and TW_1,50_, forming a polydisperse emulsion with a dominant peak at approximately 50 μm.

The emulsions and FEBs stabilized by TW exhibited a sharp increase in absolute zeta potential values immediately upon transition from the gastric to intestinal phase (Figure 5a). Furthermore, in all samples, the absolute zeta potential values increased with longer digestion times. The zeta potential range after 120 min of intestinal digestion was −47.5 to −74.9 mV. The zeta potentials of the emulsions and FEBs during intestinal digestion can be found in the Supplementary Materials (Table S4).

The emulsions and FEBs stabilized by TWCP lost their CP-induced aggregation within 5 min of intestinal digestion (Figure 3(b1,b2)). Similarly to TW, monodisperse emulsions stabilized by TWCP lost their monodisperse distribution within 5 min of digestion (Figure 6(b1–b3)). Although TWCP_1,10_ and TWCP_1,50_ lost their monodisperse distribution at each peak after 5 min, they retained their independent droplet size distributions. However, as digestion progressed, these distributions became polydisperse.

The zeta potential transition of the TWCP-stabilized emulsions and FEBs during intestinal digestion exhibited trends similar to those of the TW-stabilized samples (Figure 5b). The zeta potential range after 120 min of intestinal digestion was −49.9 to −81.8 mV.

#### 3.3.2. Analysis of Droplet Stability During Small Intestinal Digestion

The emulsions and FEBs that transitioned into the intestinal phase underwent rapid digestion by lipases and bile salts, losing both their monodisperse and independent biphasic distributions regardless of the presence of CP. The particle size distributions immediately after intestinal digestion exhibited peaks above 100 μm (Figure 6a,b). The digestive products formed upon intestinal digestion likely included various mixtures, such as undigested fat droplets, vesicles, micelles, and calcium soaps [29]. Micrographs and visual appearances taken at different digestion times revealed the presence of large oil droplets and white precipitates at 5 min after digestion, suggesting that the coarse peaks observed in the particle size distributions were due to destabilized oil droplets or aggregated materials (Figure 3 and Figure S2). The final particle size distributions after 120 min of intestinal digestion showed no significant differences between emulsions stabilized by the different emulsifiers. This can be explained by the fact that aggregation was not observed in the TWCP-stabilized emulsions and FEBs during the intestinal phase. Specifically, as soon as the emulsions transitioned from the gastric to the intestinal phase, the weak crosslinked structure formed by CP was disrupted, allowing for the rapid displacement of emulsifiers by bile salts and subsequent lipid hydrolysis by lipases.

The emulsions and FEBs stabilized by TW or TWCP exhibited a sharp increase in the absolute zeta potential values immediately upon transition from the gastric to intestinal phase (Figure 5a,b). Furthermore, in all samples, the absolute zeta potential values increased with longer digestion times. This trend was consistent with previous studies on the intestinal digestion of emulsions stabilized by nonionic emulsifiers [34,35]. This phenomenon is attributed to the presence of various anionic particles in digestive products, such as undigested fat droplets, micelles, and vesicles [29]. As negatively charged lipid digestion products, including bile salts, phospholipids, and FFAs, increased over the course of digestion, the zeta potential became increasingly negative.

The final absolute zeta potential values measured after intestinal digestion were higher in TWCP than in TW. This can be attributed to the deprotonation of carboxyl groups abundant in CP as the pH increased from the gastric to the intestinal phase, resulting in negatively charged carboxylate ions (COO^−^) [36].

### 3.4. Release of Free Fatty Acids During Small Intestinal Digestion

#### 3.4.1. Secular Changes in Free Fatty Acid Release

**Figure 7:**
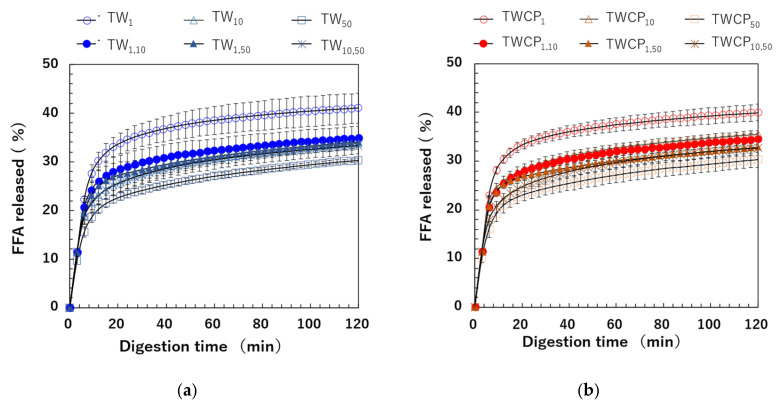
Free fatty acid (FFA) release rate at each digestion time point during in vitro intestinal digestion. (**a**) Emulsions and Food Emulsion Blends (FEBs) stabilized by Tween 20 (TW); (**b**) emulsions and FEBs stabilized by TW and citrus pectin (TWCP).

The FFA release rate was calculated based on the amount of NaOH used to maintain the intestinal digestion pH at 7.0 using Equation (3). The FFA release rates over digestion time measured for emulsions and FEBs stabilized by TW or TWCP are shown in Figure 7a,b. In all samples, FFAs were rapidly released within the first 6 min of digestion, followed by a gradual increase until the end of the digestion period. Differences in FFA release rates due to droplet size combinations emerged between the 6 min ingestion initiation period. Among the emulsions stabilized by each emulsifier, TW_1_ and TWCP_1_ exhibited the highest extent of digestion, whereas TW_50_ and TWCP_50_ showed the most suppressed lipid digestion. The digestion curves of FEBs fell within this range, indicating that the FFA release rate at each digestion time point varied depending on the droplet size combination used. The final FFA release rates ($\varphi_{max}$) after 120 min of digestion for the TW- and TWCP-stabilized emulsions were in the ranges of 30.3–41.0% and 30.3–39.9%, respectively. When comparing the same droplet size type and combination, the $\varphi_{max}$ value was slightly lower in the emulsions and FEBs containing CP than in those stabilized solely by TW, indicating the suppression of lipid digestion.

#### 3.4.2. Initial Lipid Digestion Rate

**Figure 8:**
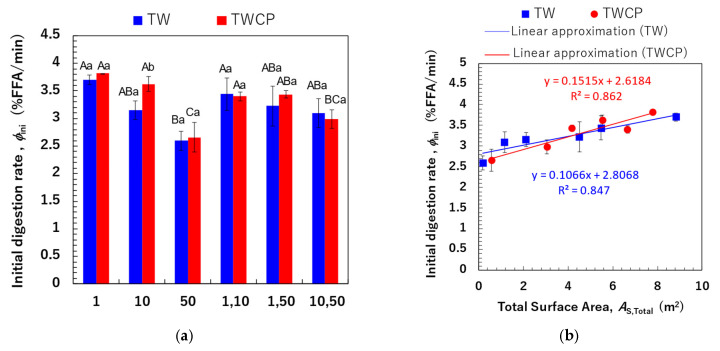
The initial digestion rate ($\varphi_{ini}$, % free fatty acid [FFA]/min) of the emulsions and Food Emulsion Blends (FEBs) and its correlation with the total droplet surface area. (**a**) The initial digestion rate of the emulsions and FEBs stabilized by Tween 20 (TW) (■) or TW and citrus pectin (TWCP) (■). The initial digestion rate was calculated based on the FFA release rate 6 min after the start of digestion. (**b**) The correlation between the total droplet surface area (m^2^) calculated based on the initial particle size distribution and the initial digestion rate. (■) Emulsions and FEBs stabilized by TW; (●) emulsions and FEBs stabilized by TWCP. Samples labeled with different uppercase letters (A, B, C) indicate significant differences in the same emulsifier with different droplet size combinations (Tukey, *p* < 0.05). Samples labeled with different lowercase letters (a, b) indicate significant differences between different emulsifiers (same droplet size range) (*t*-test, *p* < 0.05).

We determined the initial FFA release rate per unit time ($\varphi_{ini}$, % FFA/min) based on the FFA release rates measured within the first 6 min, during which FFA release exhibited a linear increase (Figure 8a). The initial digestion rates ranged from 2.59 to 3.70% FFA/min for the TW-stabilized samples and from 2.66 to 3.82% FFA/min for the TWCP-stabilized samples. A statistical analysis revealed no significant differences in $\varphi_{ini}$ values between emulsifiers and a slightly higher value for the TWCP-stabilized samples. Multiple factors, including emulsion viscosity and the type and concentration of emulsifiers and polysaccharides used, influence the initial FFA release rate [37]. In the present study, the concentration of TW in TWCP-stabilized emulsions was 0.5% (*v*/*v*), which was lower than that in the emulsions stabilized solely by TW. Additionally, viscosity measurements confirmed that emulsions stabilized by TWCP had a higher viscosity than those stabilized by TW alone due to CP addition (Figure S2). These results suggest that the opposing effects of increased digestion efficiency due to a lower TW concentration and the digestion-suppressing effect of CP did not lead to substantial differences in the initial FFA release rate.

Comparisons within the same emulsifier system revealed that emulsions with smaller droplet sizes exhibited higher initial digestion rates. To further investigate this, we calculated the total interfacial surface area ($A_{s,Total}$, m^2^) within the system and analyzed its correlation with the $\varphi_{ini}$ (Figure 8b). The $A_{s,Total}$ values for monodisperse emulsions and FEBs at the time of preparation were determined using the following equations:

(4) $$A_{s,Total}=\sum \frac{{6V}_{i}}{{\;d}_{i}}$$

(5) $$A_{s,Total}=\frac{\left(A_{s,Total1}+A_{s,Total\;2}\right)}{2}$$

where $V_{i}\;$ represents the oil volume for each droplet size (m^3^),$\;d_{i}$ is the droplet diameter (m), and $A_{s,Total1}$ and $A_{s,Total2}$ are the total droplet surface areas of the two monodisperse emulsions before mixing. Different calculation methods were employed to determine the total droplet surface area of monodisperse emulsions and FEBs. Equation (4) represents the total droplet surface area calculated based on the particle size distributions of the monodisperse emulsions. In contrast, FEBs possess two independent droplet size distributions; thus, directly calculating $A_{s,Total}$ from the mean droplet size is difficult. Although using the particle size distribution theoretically enables the determination of the actual total droplet surface area, FEBs containing 1 μm droplets exhibited an additional peak in the submicron region that was not observed in the particle size distribution of monodisperse emulsions prior to mixing (Figure 3). This peak in the submicron region significantly affected the total droplet surface area of FEBs, making accurate analysis difficult. Therefore, assuming that the two monodisperse emulsions were present in equal proportions, we calculated the total droplet surface area of the FEBs based on the $A_{s,Total}$ values determined with Equation (4) for monodisperse emulsions using Equation (5). The results shown in Figure 8b indicate a strong positive correlation between the $A_{s,Total}$ and $\varphi_{ini}$ values for both emulsifiers. The correlation coefficients (R^2^) between the $A_{s,Total}$ and $\varphi_{ini}$ values for TW and TWCP were 0.847 and 0.862, respectively. These findings suggest that FEBs potentially regulate the initial lipid digestion rate.

#### 3.4.3. Relationship Between Total Droplet Surface Area and Total Free Fatty Acid Release

**Figure 9:**
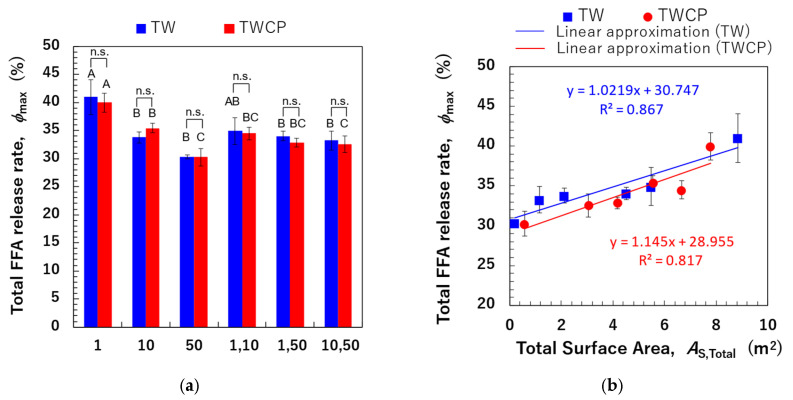
The total free fatty acid (FFA) release rate ($\varphi_{max}$, %) of the emulsions and Food Emulsion Blends (FEBs) and its correlation with the total droplet surface area. (**a**) The total FFA release rate of the emulsions and FEBs stabilized by Tween 20 (TW) (■) or TW and citrus pectin (TWCP) (■); (**b**) the correlation between the total droplet surface area ($A_{s,Total}$, m^2^) calculated based on the initial particle size distribution and $\varphi_{max}$. (■) The emulsions and FEBs stabilized by TW; (●) the emulsions and FEBs stabilized by TWCP. Samples labeled with different uppercase letters (A, B, C) indicate significant differences in the same emulsifier with different droplet size combinations (Tukey, *p* < 0.05). n.s. denotes no significant difference within the same droplet size and combination, as measured using Student’s *t*-test.

The $\varphi_{max}$ values of monodisperse emulsions and FEBs are shown in Figure 9a. A significant difference in the FFA release rate after 120 min of small intestinal digestion was observed depending on the droplet size and combination of FEB sizes. However, no significant difference in $\varphi_{max}$ values was found between samples with and without CP addition. The correlation between $A_{s,Total}$ and $\varphi_{max}$ values is shown in Figure 9b. In the Supplementary Material, additional correlations were investigated by calculating the total droplet surface area not only based on the initial particle size distribution but also using the initial $d_{4,3}$ values, the particle size distribution after gastric digestion, and $d_{4,3}$ values after gastric digestion (Figure S3). The results in Figure 9b show a strong positive correlation (R^2^ = 0.867) between the pre-digestion $A_{s,Total}$ and $\varphi_{max}$ values for TW-stabilized emulsions and FEBs. This suggests that emulsions and FEBs stabilized by highly emulsifying TW reach the small intestine without droplet aggregation, allowing lipases easier access to the oil droplet interface.

This finding implies that adjusting the combination of droplet sizes can modify the total droplet surface area within the system, even when maintaining the same oil-phase volume fraction, thereby enabling precise control over digestibility.

A strong positive correlation (R^2^ = 0.817) was observed between the $A_{s,Total}$ and $\varphi_{max}$ values for samples stabilized by the TWCP-mixed emulsifier system. This result indicates that the addition of a low concentration of CP functioned as a thickening polysaccharide, increasing viscosity and stabilizing droplets from the initial to the gastric phase. However, during transition to the intestinal phase, the crosslinked structure rapidly decomposes, allowing bile salts to replace emulsifiers and providing lipases access to the interface without hindrance, thereby facilitating lipid hydrolysis. The type, concentration, and degree of methyl esterification of pectins have been previously reported to affect emulsion digestion in the small intestine [28,38]. Future studies should thus investigate how modifying the type and concentration of added pectin can improve the control of digestibility.

Overall, these findings provide valuable insights into novel food design with controlled digestibility. However, this study had some limitations. Analysis based on droplet surface area may be restricted to emulsifiers and polysaccharides that do not cause aggregation during the small intestinal phase. In systems containing proteins or other components prone to aggregation, a detailed structural analysis of aggregates and advanced modeling approaches are necessary. In addition, many aspects of the physical and chemical properties of FEBs remain unexplored. Compared with monomodal emulsions, FEBs may exhibit unique characteristics. Therefore, future research should focus on a detailed investigation of the physical and chemical properties of FEBs, such as their storage and oxidative stability.

## 4. Conclusions

The objectives of this study were to fabricate FEBs with different droplet sizes and compositions and to elucidate their in vitro gastric and intestinal digestive characteristics. FEBs were prepared by mixing equal amounts of monodisperse emulsions produced via premix membrane emulsification, and the effects that droplet size combinations and emulsifier types have on digestive properties were then investigated. Using emulsifier solutions of either TW or the mixed emulsifier system (TWCP), FEBs with two independent droplet size distributions were successfully prepared.

The in vitro gastric and intestinal digestion experiments demonstrated that all FEBs maintained independent droplet size distributions until the gastric digestion stage. During the intestinal phase, bimodal distribution was lost in all samples. However, varying the droplet size combination altered the FFA release rate during intestinal digestion and the final FFA release percentage after 120 min. These digestion parameters exhibited a strong correlation with the total droplet surface area at the time of fabrication. This finding suggests that FEBs enable easy adjustment of the total droplet surface area within the system, offering a potential approach for precise control over lipid digestion. Additionally, a positive correlation between the droplet surface area and FFA release rate after small intestinal digestion was observed even in complex systems with the addition of low concentrations of CP. This result suggests the potential to control lipid digestibility during small intestinal digestion.

Potential applications of FEBs include the development of jelly-based foods for the elderly and nutritionally controlled functional foods. Furthermore, as droplet size and distribution influence taste, texture, and aroma perception, FEBs could be utilized for the development of novel foods with controlled sensory and textural properties.

## Figures and Tables

**Table 1:** Composition and characteristics of defined emulsions and Food Emulsion Blends.

	Continuous Phase	Dispersed Phase	Membrane Pore Size
TW_1_	1.0% (*w*/*w*) Tween 20 (TW)	10% (*v*/*v*) soybean oil	1 μm
TW_10_			10 μm
TW_50_			50 μm
TW_1,10_			1 μm, 10 μm
TW_1,50_			1 μm, 50 μm
TW_10,50_			10 μm, 50 μm
TWCP_1_	0.5% (*w*/*w*) Tween 20, 0.5% (*w*/*w*) citrus pectin (TWCP)	10% (*v*/*v*) soybean oil	1 μm
TWCP_10_			10 μm
TWCP_50_			50 μm
TWCP_1,10_			1 μm, 10 μm
TWCP_1,50_			1 μm, 50 μm
TWCP_10,50_			10 μm, 50 μm

**Table 2:** Compositions of simulated gastric and small intestinal fluids.

Simulated Gastric Fluid			Simulated Small Intestinal Fluid		
KCl	0.514	g/L	CaCl_2_(H_2_O)_2_	2.9	g/L
KH_2_PO_4_	0.122	g/L	Sodium taurodeoxycholate	5	g/L
NaHCO_3_	2.1	g/L	Lipase *	2000	U/mL
NaCl	2.76	g/L			
MgCl_2_(H_2_O)_6_	0.02	g/L			
(NH_4_)_2_CO_3_	0.074	g/L			
CaCl_2_(H_2_O)_2_ *	0.022	g/L			
HCl	49.8	mM			
Pepsin *	4000	U/mL			

* Added just before beginning the experiment.

## Data Availability

Data will be made available upon request from the corresponding author.

## Supplementary Materials

The following supporting information can be downloaded at: https://www.mdpi.com/article/10.3390/foods14071239/s1, Reference [39] are cited in the Supplementary Materials. Table S1: The compositions of the emulsifiers and dispersed phases used in this study; Table S2: The physical properties of the continuous and dispersed phases used in this study; Table S3: The mean droplet sizes at the initial phase and at each digestion phase; Table S4: The zeta potential at the initial phase and at each digestion phase; Figure S1: The viscosity of the emulsions and Food Emulsion Blends stabilized by Tween 20 aqueous solution (TW) and a mixed solution of TW and citrus pectin (TWCP); Figure S2: The visual appearance of the emulsions and Food Emulsion Blends at different digestion stages; Figure S3: The correlation between the total droplet surface area and free fatty acid (FFA) release rate after 120 min of digestion.
