# Supplementary material for: The Influence of Droplet Size and Emulsifiers on the In Vitro Digestive Properties of Bimodal Oil-in-Water Emulsions
Source: Foods. 2025 Apr 1;14(7):1239. doi: 10.3390/foods14071239 (PMC11988771; doi:10.3390/foods14071239)
Supplement: Supplementary file 1 [file foods-14-01239-s001.zip › foods-3529653-supplementary.pdf]

*Supplementary materials*

## S1. Physical properties of the continuous and dispersed phases

The composition of the emulsifiers and dispersed phase used in this study is presented in Table S1. For the emulsifiers, molecular weight and hydrophilic–lipophilic balance (HLB) values are provided. The HLB value is an index that represents the balance between hydrophilic and lipophilic properties of a surfactant, where higher values indicate greater hydrophilicity and lower values indicate greater lipophilicity. Additionally, the fatty acid composition of soybean oil used as the dispersed phase is included. Physical properties of the continuous and dispersed phases used in this study are shown in Table S2. The viscosity, density, and interfacial tension of the continuous and dispersed phases were measured. Viscosity was determined using a tuning-fork vibro-viscometer (SV-10 model; A&D Co., Ltd., Tokyo, Japan). A small sample container was used, and the average of three measurements recorded. All measurements were conducted at 25 °C. The density of the samples was measured using a density meter (DA-130N; KEM Co., Ltd., Kyoto, Japan). The interfacial tension was measured to evaluate the reduction effect of each emulsifier using a fully automatic interfacial tensiometer (DM-301; Kyowa Interface Science Co., Ltd., Niiza, Japan) with the pendant drop method. Images of the maximum drop size of each continuous phase in rice bran oil were captured, and the interfacial tension calculated using the image analysis software, FAMAS (v.5.1.1). The interfacial tension of each continuous phase drop was measured 10 times immediately before detachment from the needle tip, and the average value calculated.

Table S1. Composition of the emulsifiers and dispersed phase used in this study. M.W., molecular weight; HLB, hydrophilic–lipophilic balance.

| Emulsifiers | | | Dispersed phase | | |
|---|---|---|---|---|---|
| | M.W. | HLB | | Main component | Main composition [1] |
| Tween 20 | 1228 | 16.7 | Soybean oil | Long-chain Triglyceride | 56% Linoleic acid (C18:2)<br>24% Oleic acid (C18:1) |

Table S2. Physical properties of the continuous and dispersed phases used in this study, including viscosity ($\eta$, mPa·s), density ($\rho$, kg·m$^{-3}$), and interfacial tension ($\gamma$, mN·m$^{-1}$).TW, Tween 20; TWCP, Tween 20 and citrus pectin.

| | | Viscosity, $\eta$ (mPa s) | Density, $\rho$ (kg m$^{-3}$) | Interfacial tension, $\gamma$ (mN m$^{-1}$) |
|---|---|---|---|---|
| Continuous phase | TW | 1.07 | 998.2 | 5.01 |
| | TWCP | 2.07 | 830.0 | 7.00 |
| Dispersed phase | Soybean oil | 48.70 | 920.0 | – |

## S2. Mean droplet size of the emulsions and food emulsion blends

The mean droplet sizes at the initial phase and at each digestion phase are presented in Table S3. Measurements were conducted independently three times, and the average values used.

Table S3. Mean droplet sizes at the initial phase and at each digestion phase.TW, Tween 20; TWCP, Tween 20 and citrus pectin.

| | Initial phase | Gastric phase | Small intestine_ 5 min | Small intestine_ 20 min | Small intestine_ 60 min | Small intestine_ 120 min |
|---|---|---|---|---|---|---|
| $TW_{1}$ | 1.05 ± 0.08 | 0.88 ± 0.22 | 1.64 ± 0.68 | 1.79 ± 1.12 | 3.11 ± 2.39 | 1.81 ± 0.71 |
| $TW_{10}$ | 10.98 ± 1.56 | 9.23 ± 2.97 | 110.32 ± 59.45 | 78.12 ± 29.96 | 60.35 ± 16.77 | 13.90 ± 8.88 |
| $TW_{50}$ | 51.99 ± 5.71 | 39.14 ± 10.42 | 118.36 ± 58.73 | 97.85 ± 34.52 | 57.97 ± 3.43 | 38.44 ± 14.53 |
| $TW_{1,10}$ | 4.86 ± 0.42 | 5.45 ± 0.75 | 118.36 ± 88.12 | 48.10 ± 47.30 | 28.43 ± 15.01 | 21.48 ± 6.08 |
| $TW_{1,50}$ | 23.63 ± 7.83 | 18.13 ± 1.86 | 50.18 ± 47.73 | 35.40 ± 37.26 | 31.10 ± 21.35 | 19.87 ± 9.33 |
| $TW_{10,50}$ | 25.64 ± 0.48 | 23.81 ± 2.14 | 83.44 ± 67.79 | 105.09 ± 37.96 | 50.48 ± 5.75 | 43.42 ± 22.36 |
| $TWCP_{1}$ | 1.19 ± 0.06 | 1.22 ± 0.04 | 7.81 ± 8.57 | 5.95 ± 4.63 | 21.18 ± 17.97 | 4.44 ± 2.55 |
| $TWCP_{10}$ | 11.37 ± 0.79 | 12.31 ± 0.25 | 62.66 ± 55.21 | 28.09 ± 11.27 | 67.35 ± 19.48 | 48.20 ± 36.42 |
| $TWCP_{50}$ | 46.94 ± 2.65 | 45.59 ± 1.58 | 114.75 ± 96.69 | 94.29 ± 72.60 | 75.99 ± 23.69 | 55.11 ± 37.47 |
| $TWCP_{1,10}$ | 4.88 ± 0.54 | 4.77 ± 0.60 | 114.75 ± 5.51 | 18.70 ± 6.15 | 25.31 ± 14.24 | 15.67 ± 7.22 |
| $TWCP_{1,50}$ | 21.32 ± 2.84 | 19.23 ± 0.20 | 54.09 ± 40.12 | 26.37 ± 8.56 | 17.32 ± 1.65 | 21.83 ± 3.03 |
| $TWCP_{10,50}$ | 27.50 ± 1.31 | 27.49 ± 1.21 | 115.62 ± 103.44 | 15.94 ± 5.91 | 67.16 ± 20.16 | 46.63 ± 33.87 |

**S3. Zeta potential of the emulsions and food emulsion blends**

The zeta potentials at the initial stage and at each digestion stage are presented in Table S4. Measurements were conducted independently three times, and the average values used.

Table S4. Zeta potential at the initial phase and at each digestion phase.TW, Tween 20; TWCP, Tween 20 and citrus pectin.

| | Initial phase | Gastric phase | Small intestine_ 5 min | Small intestine_ 20 min | Small intestine_ 60 min | Small intestine_ 120 min |
|---|---|---|---|---|---|---|
| TW$_{1}$ | −37.9 ± 1.4 | −11.5 ± 0.4 | −51.0 ± 3.5 | −61.2 ± 5.1 | −65.7 ± 7.5 | −74.9 ± 9.5 |
| TW$_{10}$ | −58.0 ± 13.3 | −7.2 ± 2.2 | −35.3 ± 3.1 | −42.9 ± 3.5 | −50.5 ± 5.7 | −56.9 ± 5.0 |
| TW$_{50}$ | −42.0 ± 12.5 | −2.8 ± 0.5 | −37.0 ± 3.4 | −39.9 ± 1.3 | −43.4 ± 2.9 | −48.0 ± 4.4 |
| TW$_{1,10}$ | −39.6 ± 0.8 | −7.1 ± 0.4 | −43.9 ± 3.8 | −47.9 ± 5.9 | −50.4 ± 3.5 | −57.8 ± 4.8 |
| TW$_{1,50}$ | −35.5 ± 3.5 | −5.4 ± 2.1 | −40.5 ± 3.4 | −40.4 ± 4.7 | −43.9 ± 4.8 | −47.5 ± 9.2 |
| TW$_{10,50}$ | −47.0 ± 10.9 | −3.4 ± 0.3 | −33.7 ± 3.1 | −42.8 ± 2.7 | −49.0 ± 5.2 | −53.2 ± 6.4 |
| TWCP$_{1}$ | −34.0 ± 8.7 | −11.0 ± 3.4 | −53.1 ± 5.2 | −67.4 ± 4.4 | −74.5 ± 5.9 | −81.8 ± 3.3 |
| TWCP$_{10}$ | −17.6 ± 3.3 | −6.9 ± 1.2 | −40.3 ± 4.8 | −47.8 ± 2.0 | −52.6 ± 6.7 | −59.6 ± 7.8 |
| TWCP$_{50}$ | −10.6 ± 2.5 | −5.3 ± 1.3 | −38.0 ± 3.7 | −44.7 ± 3.0 | −48.7 ± 2.2 | −49.9 ± 3.8 |
| TWCP$_{1,10}$ | −18.8 ± 0.9 | −13.0 ± 7.7 | −40.7 ± 7.4 | −49.1 ± 12.0 | −51.9 ± 10.7 | −65.6 ± 20.1 |
| TWCP$_{1,50}$ | −14.6 ± 3.4 | −9.0 ± 3.2 | −43.0 ± 5.6 | −45.6 ± 7.9 | −46.6 ± 6.3 | −51.2 ± 6.5 |
| TWCP$_{10,50}$ | −15.4 ± 4.5 | −5.2 ± 0.9 | -39.2 ± 6.3 | −47.3 ± 5.0 | −53.8 ± 2.8 | −57.8 ± 3.0 |

## S4. Viscosity of the emulsions and food emulsion blends

The viscosity of emulsions and food emulsion blends (FEBs) stabilized by Tween 20 (TW) or by TW and citrus pectin (TWCP) is shown in Figure S1. Viscosity of the oil-in-water (O/W) emulsions was measured using a viscometer (Brookfield DV-II+ Pro; AMETEK Brookfield, Middleboro, MA, USA). A volume of 6.7 mL O/W emulsion was placed into the chamber, and the spindle (SC4-18, diameter: 17.5 mm, height: 31.7 mm) rotated at 1–40 rpm. The viscosity value was obtained and recorded 30 s after the spindle began rotating. As emulsion viscosity is temperature-sensitive, a water-circulating thermostatic bath was used to maintain the sample temperature at 25 °C. Each measurement was performed in triplicate, and the average value used for analysis.

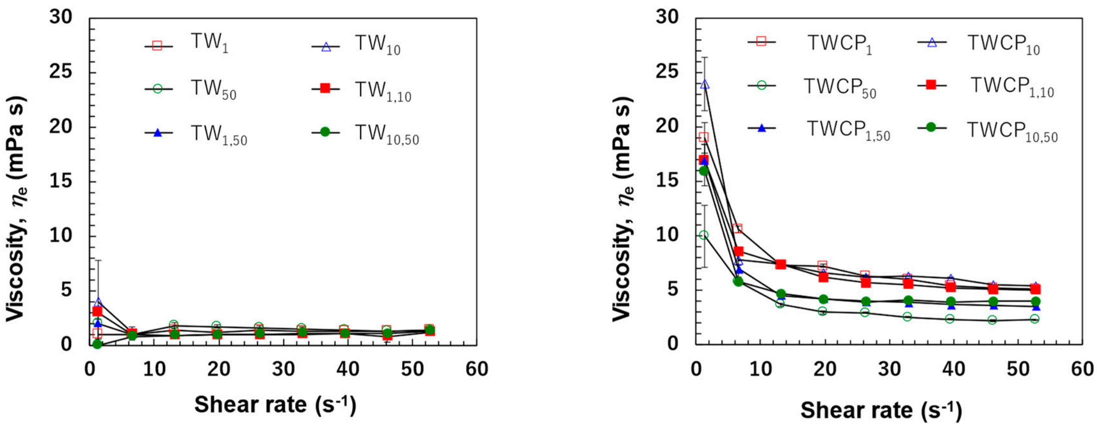


(a) (b)

Figure S1. Viscosity of the emulsions and food emulsion blends stabilized by (a) Tween 20 (TW) and (b) TW and citrus pectin (TWCP).

**S5. Visual appearance of the emulsions and food emulsion blends at different digestion stages**

Figure S2 shows the visual appearance of samples at different digestion stages, including immediately after preparation, during gastric digestion, and during intestinal digestion (5, 20, 60, and 120 min). Samples (3 mL) were collected and placed into vials for imaging.

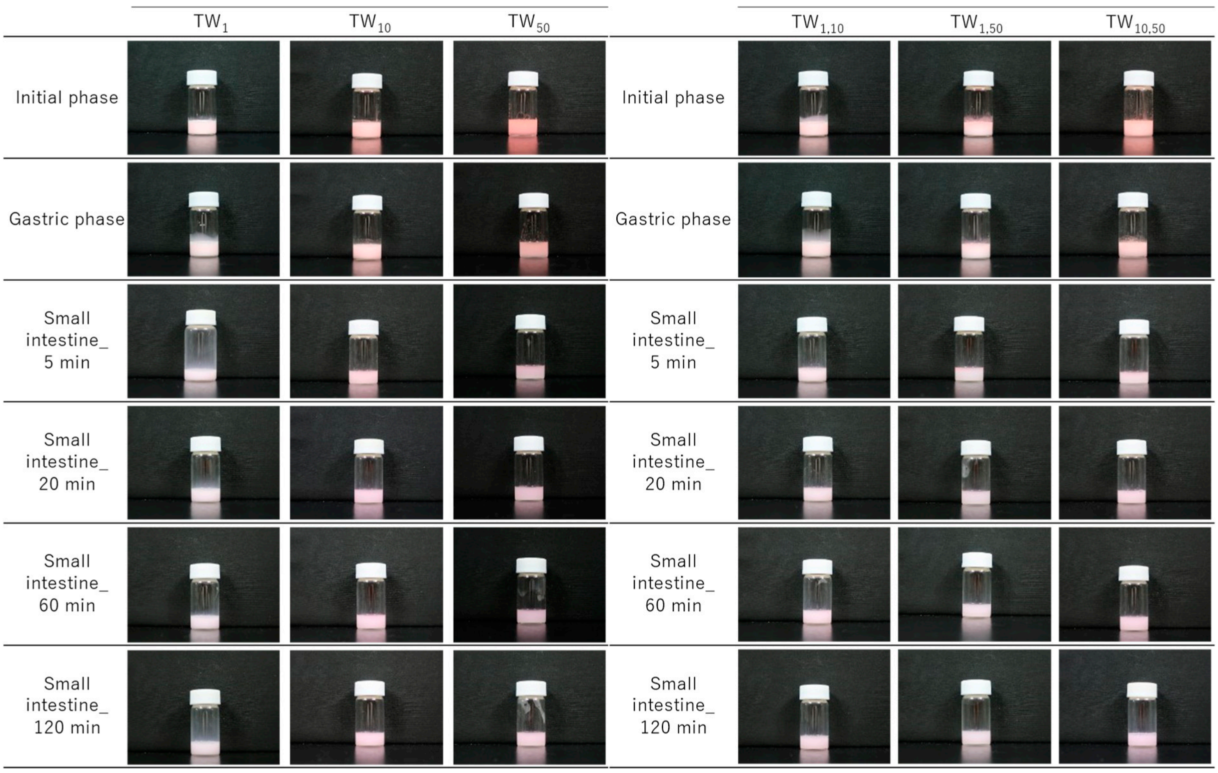


(a1) (a2)

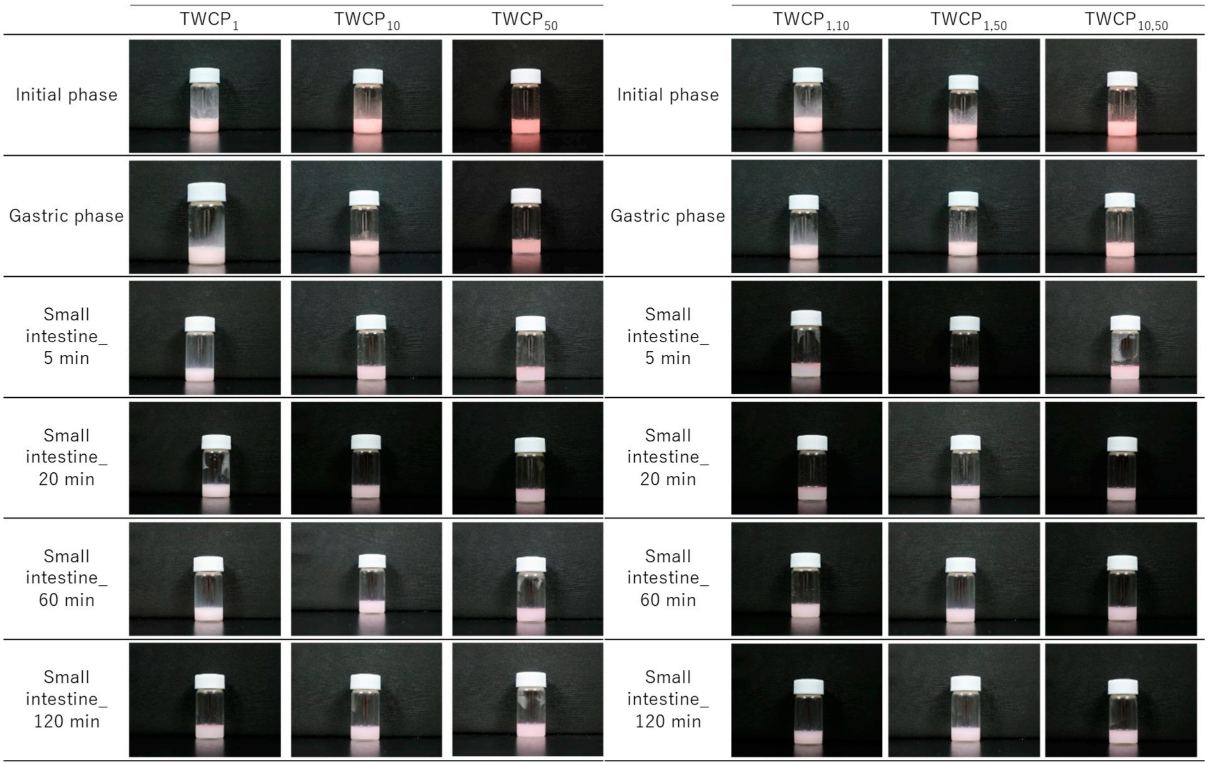


(b1) (b2)

Figure S2. Visual appearance of the emulsions and food emulsion blends at different digestion stages: (a1) $TW_1$, $TW_{10}$, and $TW_{50}$; (a2) $TW_{1,10}$, $TW_{1,50}$, and $TW_{10,50}$; (b1) $TWCP_1$, $TWCP_{10}$, and $TWCP_{50}$; (b2) $TWCP_{1,10}$, $TWCP_{1,50}$, and $TWCP_{10,50}$. TW, Tween 20; TWCP, Tween 20 and citrus pectin.

## S6. Correlation between total droplet surface area and free fatty acid release after 120 min of digestion

Figure S3 shows the correlation between the total droplet surface area at different digestion stages and free fatty acid release rate after 120 min of digestion ($\varphi_{max}$, %). The total droplet surface area of the samples immediately after preparation and after gastric digestion was calculated based on particle size distribution or the mean droplet diameter. The total droplet surface area based on the mean droplet diameter at the initial stage ($A_{\text{Initial},d}$, m²) was calculated using the following equations:

$$A_{\text{Initial},d} = \frac{6V_o}{d_{Initial}} \quad \text{(Eq. S1-1)}$$

$$A_{\text{Initial},d} = \frac{(A_{\text{Initial},d1} + A_{\text{Initial},d2})}{2} \quad \text{(Eq. S1-2)}$$

where $V_o$ represents the volume of the dispersed phase (m³); $d_{Initial}$ the mean droplet diameter immediately after preparation; and $A_{s,Total1}$ and $A_{s,Total2}$ the total droplet surface areas of the two monodisperse emulsions before mixing. Different calculation methods were employed to determine the total droplet surface area of monodisperse emulsions and FEBs. Eq. S1-1 was used to calculate the total droplet surface area of the monodisperse emulsions immediately after preparation. However, as Eq. S1-1 cannot be applied to calculate the droplet surface area of FEBs with a bimodal distribution, Eq. S1-2 was used instead.

Subsequently, the total droplet surface area based on the mean droplet diameter after gastric digestion ($A_{\text{Gastric},d}$, m²) and based on the particle size distribution after gastric digestion ($A_{Gastric,dis}$, m²) were calculated using the following equations:

$$A_{\text{Gastric},d} = \frac{6V_o}{d_{Gastric}} \quad \text{(Eq. S2-1)}$$

$$A_{\text{Gastric},d} = \frac{(A_{\text{Gastric},d1} + A_{\text{Gastric},d2})}{2} \quad \text{(Eq. S2-2)}$$

$$A_{Gastric,dis} = \sum \frac{6V_{i,Gastric}}{d_{i,Gastric}} \quad \text{(Eq. S3-1)}$$

$$A_{Gastric,dis} = \frac{(A_{Gastric,dis1} + A_{Gastric,dis2})}{2} \quad \text{(Eq. S3-2)}$$

where $d_{Gastric}$ represents the mean droplet diameter after the gastric digestion stage; $A_{\mathrm{Gastric},d1}$ and $A_{\mathrm{Gastric},d2}$ the total droplet surface areas based on the mean droplet diameters of the two monodisperse emulsions after gastric digestion; $V_{i,Gastric}$ the oil volume for each droplet size after gastric digestion (m[3]); $d_{i,Gastric}$ the droplet diameter after gastric digestion (m); and $A_{Gastric,dis1}$ and $A_{Gastric,dis2}$ the total droplet surface areas based on the particle size distributions of the two monodisperse emulsions after gastric digestion (Figure S3b,c).

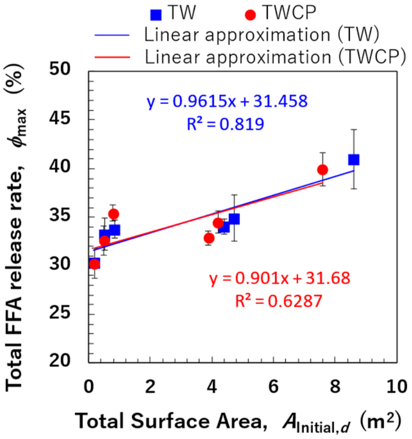


(a)

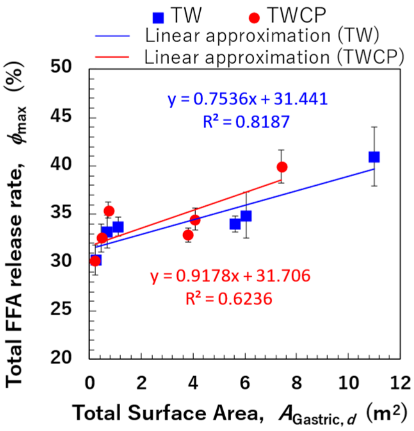


(b)

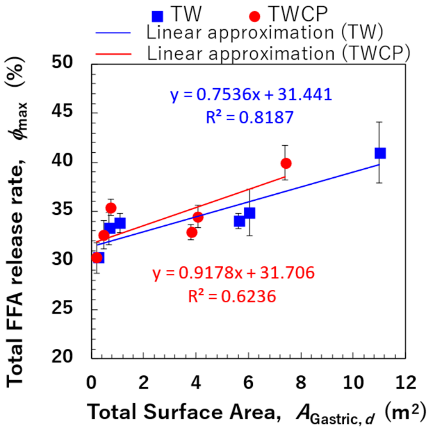


(c)

Figure S3. Correlation between the total droplet surface area (m[2]) calculated using different digestion stages and calculation methods and the free fatty acid (FFA) release rate after small intestinal digestion ($\varphi_{max}$): (a) total droplet surface area calculated based on the initial mean droplet size ($A_{\mathrm{Initial},d}$); (b) total droplet surface area calculated based on the mean droplet size after gastric digestion ($A_{\mathrm{Gastric},d}$); (c) total droplet surface area alculated based on the droplet size distribution after gastric digestion ($A_{\mathrm{Gastric,dis}}$). TW, Tween 20; TWCP, Tween 20 and citrus pectin.

## References


1. CHANGWATCHAI, T. Molecular Distillation for Separation of Fatty Acids and Triglycerides, PhD Thesis, University of Tsukuba, Japan, March 2022.
